# Development of an artificial neural network as a tool for predicting the chemical attributes of fresh peach fruits

**DOI:** 10.1371/journal.pone.0251185

**Published:** 2021-07-30

**Authors:** Mahmoud Abdel-Sattar, Rashid S. Al-Obeed, Abdulwahed M. Aboukarima, Dalia H. Eshra

**Affiliations:** 1 Department of Plant Production College of Food and Agriculture Sciences, King Saud University, Riyadh, Saudi Arabia; 2 Pomology Department, Faculty of Agriculture, Alexandria University, Alexandria, Egypt; 3 Department of Agricultural Engineering, College of Food and Agriculture Sciences, King Saud University, Riyadh, Saudi Arabia; 4 Agricultural Engineering Research Institute, Agricultural Research Center, Giza, Egypt; 5 Food Science and Technology Department, Faculty of Agriculture, Alexandria University, Alexandria, Egypt; Newcastle University, UNITED KINGDOM

## Abstract

This investigation aimed to develop a method to predict the total soluble solids (TSS), titratable acidity, TSS/titratable acidity, vitamin C, anthocyanin, and total carotenoids contents using surface color values (L*, Hue and chroma), single fruit weight, juice volume, and sphericity percent of fresh peach fruit. Multiple regression analysis (MLR) and an artificial neural network (ANN) were employed. An ANN model was developed with six inputs and 15 neurons in the first hidden layer for the prediction of six chemical composition parameters. The results confirmed that the ANN model R^*2*^ = 974–0.998 outperformed the MLR models R^*2*^ = 0.473–0.840 using testing dataset. Moreover, sensitivity analysis revealed that the juice volume was the most dominating parameter for the prediction of titratable acidity, TSS/titratable acidity and vitamin C with corresponding contribution values of 39.97%, 50.40%, and 33.08%, respectively. In addition, sphericity percent contributed by 23.70% to anthocyanin and by 24.08% to total carotenoids. Furthermore, hue on TSS prediction was the highest compared with the other parameters, with a contribution percentage of 20.86%. Chroma contributed by different values to all variables in the range of 5.29% to 19.39%. Furthermore, fruit weight contributed by different values to all variables in the range of 16.67% to 23.48%. The ANN prediction method denotes a promising methodology to estimate targeted chemical composition levels of fresh peach fruits. The information of peach quality reported in this investigation can be used as a baseline for understanding and further examining peach fruit quality.

## 1. Introduction

The genus *Prunus* includes many important species of substantial economic importance, such as *Prunus persica* L. (peach), which thrive in temperate and warm regions partly due to their low chilling requirements. Peaches are esteemed as delicious and healthy summer fruit in most temperate zones globally. Several peach cultivars have been organized into three groups: clingstone peaches, peaches, and nectarines due to variation in some pomological and biological characteristics [1].

Peaches have substantial dietetic value. They contain fiber and are a rich source of vitamins, particularly vitamins A, B, and C, carbohydrates, and some mineral nutrients [2]. However, planting location, cultivar, stage of maturity, and climatic conditions influence fruit composition [3]. For peaches that are consumed fresh, fruit size is an important attribute of quality [4] since it is a requisite required by the consumer [5]. Additionally, the quality attributes that are most significant in fruits include the total soluble solids (TSS), color, titratable acidity, firmness, and TSS/titratable acidity [6–8]. Matias *et al*. [6] found strong correlations between fruit mass, suture diameter, equatorial diameter, and polar diameter as well as between carotenoids content and hue angle for pulp color in 28 peach cultivars.

The quality of peach fruits can be determined using chemical attributes, such as citric acid, carotenoids, lactones, and polyphenols [9]. Additionally, peach ripening involves complex chemical and physical changes, including color changes [10]. However, these changes affect the sensorial characteristics and nutritional value of peach fruits. Thus, statistical analyses using Pearson’s pairwise correlation and regression analysis between the variables can be used to examine variability in the chemical parameters that depict peach quality [11]. However, titratable acidity is the most substantial contributor to overall flavor and acid taste: TSS is the best predictor of sweet taste, and TSS/titratable acidity is the best predictor of fruit flavor [12]. However, obtaining chemical attributes is inexpensive as it uses simple analytical methods [13], but it destroys the fruit and is not adequate to monitor peach quality in modern grading lines, as it renders uncertain values and is highly variable, depending on external factors, such as seasonal and genetic factors, and harvest date. An alternative method is the use of non-destructive methods that are more effective than analytical methods, as non-destructive methods are principally based on physical characteristics, which correlate well with certain quality factors of fruits and vegetables [14]. At harvest time, the internal quality of peach cultivars can be determined using an alternative approach of using simple measurements, as chemical parameters of fruits require expensive equipment and time to acquire [15]. This process can be completed by finding the link between easier and lower cost measuring indices, such as surface color parameters, peach weight, and peach fruit dimensions with individual chemical composition parameters. ANN modeling offers a way of examining uneven and multi-dimensional datasets arising from data collection activities [15].

The application of ANN to the task of solving non-linear and complex systems is promising [16]. However, the advantage of ANNs modeling over conventional modeling methods lies in their skill to solve issues that do not have an algorithmic solution, or the available solution is too complex to be found. Moreover, ANNs can quickly recognize linear and non-linear patterns [17]. The ANN technique acts as a mathematical model to determine the closest finding to the expected value. For example, Furferi *et al*. [18] developed an ANN model to predict peroxide and acidity levels of olive oil during the continuous extraction process. This allows oil quality to be monitored and controlled during the extraction process in real-time. Rai *et al*. [19] applied an ANN model to predict the viscosity of clarified fruit juice for peach, orange, and pear fruits as a function of concentration and temperature. Yalcin *et al*. [20] indicated that the ANN technique can be considered an alternative to other methods utilized to determine the fatty acid composition of oils. Cimpoiu *et al*. [21] used an ANN model with the back-propagation algorithm to create a model for determining the antioxidant activity of some classes of tea, such as express black, black, and green teas. Their results indicated a correlation of 99.9% between experimental and predicted antioxidant activity. Thus, the established ANN model promoted the inspection of the antioxidant activity of tea. Huang *et al*. [22] employed ANN model to predict soluble solids, titratable acid content and the ratio of soluble solids to titratable acid in loquat fruits. The results showed that compared with the MLR model R2 = 0.6772, R2 = 0.5520 and R2 = 0.6025, respectively, the ANNs predicted soluble solids, titratable acid content and the ratio of soluble solids to titratable acid with higher accuracy and effectiveness R2 = 0.9597, R2 = 0.9580 and R2 = 0.9658, respectively.

The ANN approach is particularly useful for complicated issues involving several parameters with limited information on the interactions between variables and their variation [23]. Being able to predict the chemical composition of fruit juice without using expensive analyses is important for determining fruit quality in the fruit industry. Therefore, this investigation aimed to appraise and validate the precision of the ANN technique to predict some chemical composition parameters in peach fruits through its ability to learn complex, non-linear relationships between inputs and outputs. The obtained results provide an important contribution to this research area and the fruit industry.

## 2. Materials and methods

### 2.1. Experimental site growing conditions and plant materials

**Fig 1:**
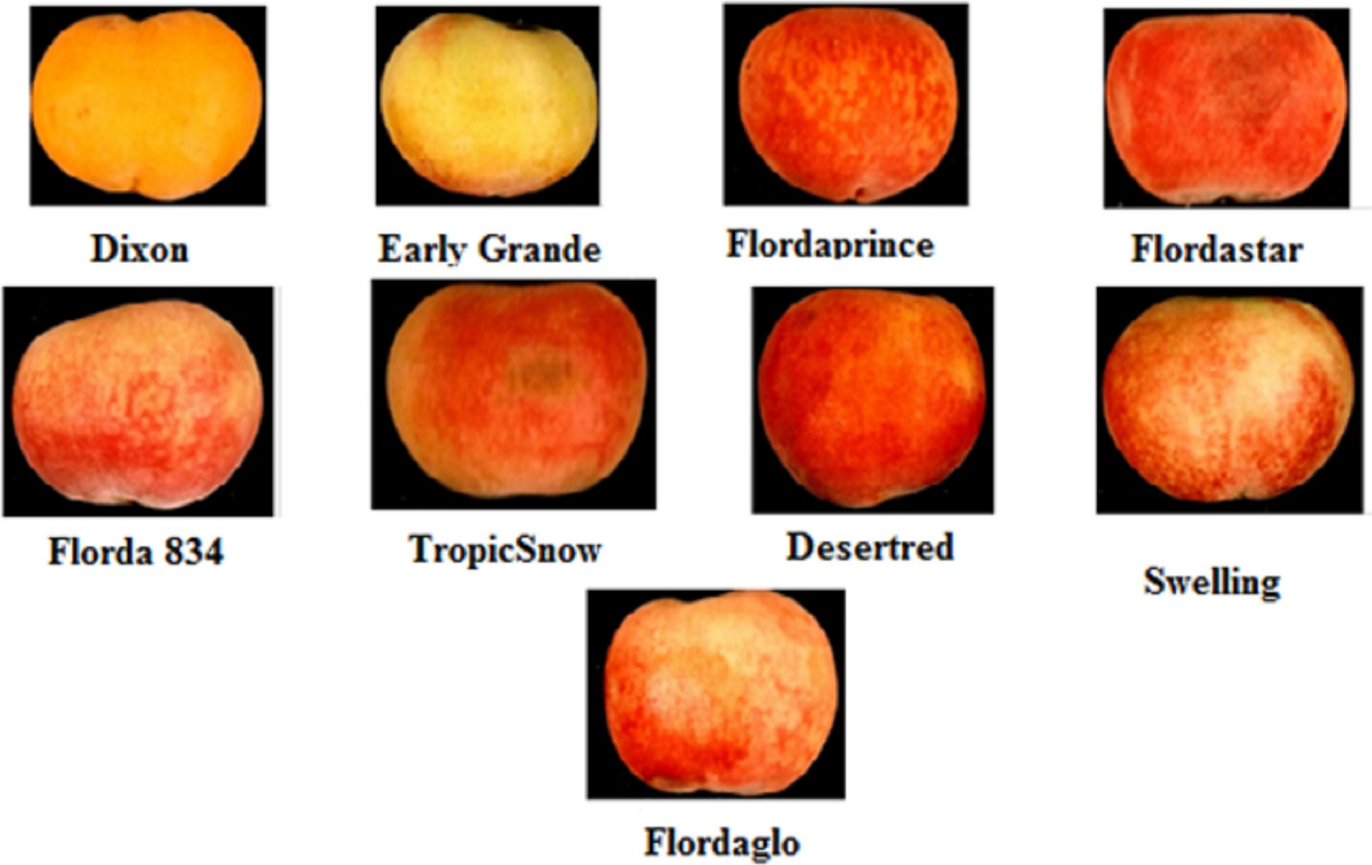
Nine peach cultivars, *Prunus persica L*.

This investigation was conducted during the 2019 growing season on nine peach cultivars (*Prunus persica* L.), namely Dixon, Early Grande, Flordaprince, Flordastar, Flordaglo, Florda 834, TropicSnow, Desertred, and Swelling, as shown in Fig 1. The peach trees were eight years old and grown in sandy soil, with a pH of 7.7–7.8, in private commercial orchards near Pico Company, El-Behera Governorate, Egypt. The peach trees were grafted on Nemaguard rootstock and spaced at 3.5 x 5 m apart, and they received standard cultural practices, including fertilization, pruning and pest and disease control. Drip irrigation was used. Four trees, which were as uniform as possible, were selected to get samples.

Fruits were harvested when they were fully mature following commercial practice, and they were immediately transported to the laboratory located at the Food Science and Technology Department, Faculty of Agriculture, Alexandria University, Alexandria, Egypt. The fruits were harvested at the ripe stage and samples with defects, such as sunburn, cracks, bruises, and cuts in the husk, were discarded. The purpose of the study was to evaluate some physical and chemical properties and to build a prediction model of the total soluble solids, titratable acidity, TSS/titratable acidity, vitamin C, anthocyanin, and total carotenoids contents of the fresh peach fruit.

### 2.2. Measurements and determinations

**Fig 2:**
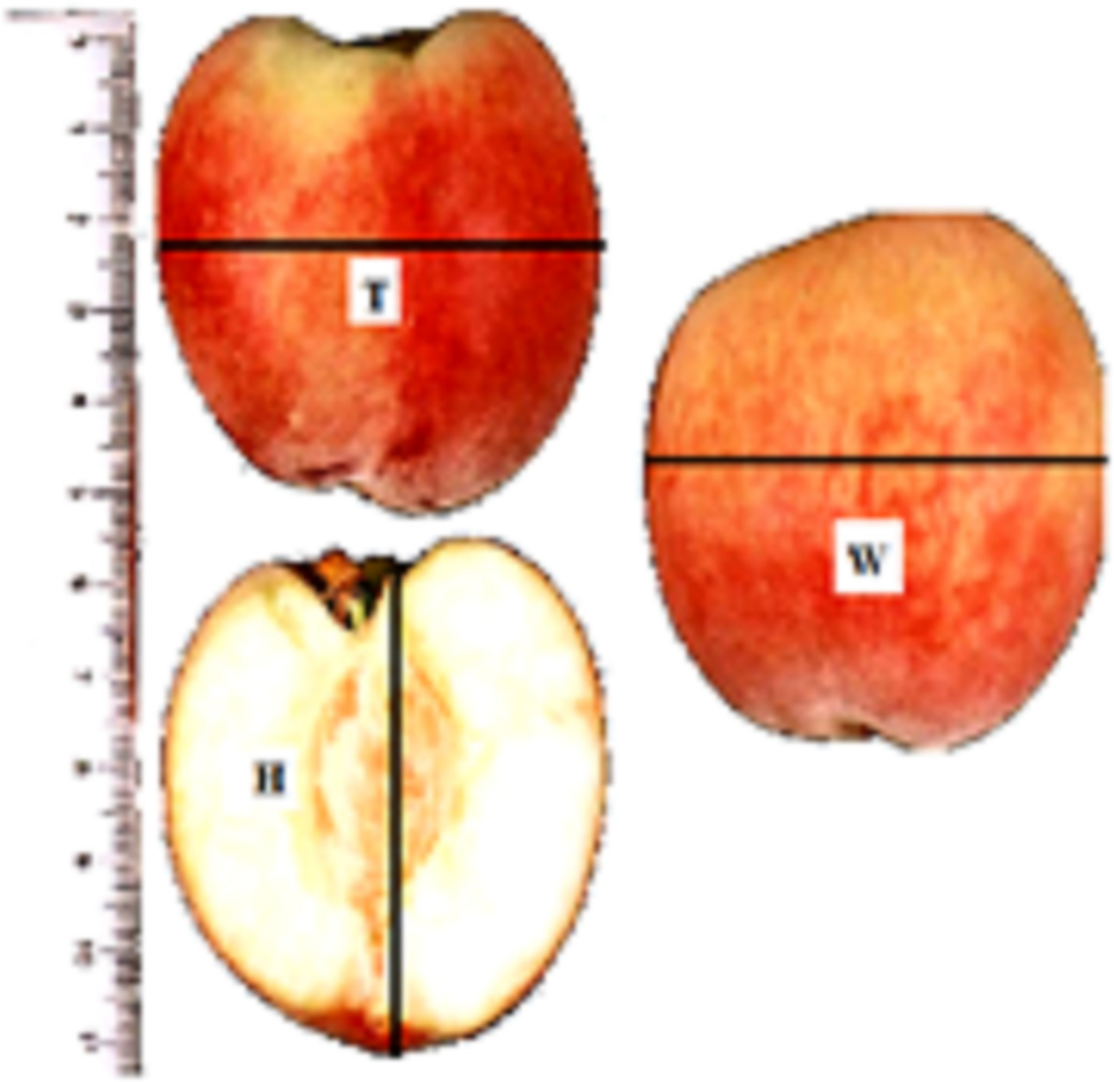
Three major dimensions of peach.

Twenty mature fruit were randomly selected from each experimental tree to determine the physical and chemical properties of the fresh fruit. Fruit weight was determined by weighing the selected samples of each cultivar using a digital weighing scale with ±0.001-gram accuracy. To determine the average size of the fruits, three linear dimensions, namely length (H); equivalent distance of the stem (head) to the calyx (bottom), width (W); the longest dimension perpendicular to H, and thickness (T); the longest dimension perpendicular to H and W, (Fig 2), were measured using a digital caliper with accuracy of 0.01 mm as described in [24]. The sphericity percent (*ϕ*, %) of the fruit is term used to express the shape of a material, and was calculated using the formula used by Vivek *et al*. [25]:

$$\phi =(\frac{{\left(H*W*T\right)}^{1/3}}{H})\times 100$$ (1)

The percentage of TSS in the fruit juice was determined using a hand refractometer, and the percentage of total acidity was determined as the amount of malic acid per 100 ml of juice, according to [13] by titration with 0.1 sodium hydroxide using phenolphthaline as an indicator. The ascorbic acid (vitamin C) content in the juice was determined by titration using 2, 6-dichlorophenolindolphenol blue dye following [13], and it was expressed as milligrams of ascorbic acid/100 millimeter juice. The anthocyanin pigment content was determined (mg/100 g fresh weight) following [26]. Total carotenoids pigments were determined according to the method of [27] and expressed as milligrams/100 g of fresh weight. The peach juice was made by squeezing fresh fruit samples after acquired their images and its volume was determined using 100 mL graduated cylinder.

We measured the color of the fresh peach fruit cultivar samples using simple digital imaging [28]. Fresh peaches were placed in a white plastic container acted as lighting box, which was illuminated with two lighting 26 W fluorescent lamps (lumen = 1250 +/-20%). All lamps, which were 13 cm long, were situated 45 cm above the sample of peaches. A high-resolution digital camera (Canon XUS105, 12.0 Megapixel, four digital zoom) was used. However, the camera mode was switched to Auto. A laptop computer was used to store images. The digital camera was located vertically over the background at a distance of 45 cm. The angle between the camera lens and the lighting source axis was approximately 90°. The camera was fixed on top of the lighting box.

Twenty peach images were captured for each cultivar. The color of the peach images was examined quantitatively with the help of Photoshop software [29] using the Histogram Window. The mean color values of lightness (L), (a), and (b) were captured. These values are not standard color values. The values of L, a, and b can be changed to L*, a*, and b* values using the formulas presented in [28]:

$$L*=\frac{\mathrm{Lightness}}{255}\times 100$$ (2)

$$a^{*}=\frac{240\;a}{255}-120$$ (3)

$$b^{*}=\frac{240\;b}{255}-120$$ (4)

L*, a*, and b*coordinate axis defines the three-dimensional CIE color space [30]. Where, L* is lightness, a* is redness, b* is yellowness. The positive values of b* show the yellow color, so, the peaches with the highest value for b* are specified as being of good quality.

All color measurements for peach cultivars were made on unwashed, unbrushed fruits. Moreover, fruit chromaticity was recorded in CIE L*, a*, and b* color space coordinates. In this system of color representation, the values L*, a*, and b* describe a uniform three-dimensional color space, *where L* corresponds to a dark/bright scale (0 = black, 100 = white), a* is negative for green and positive for red, and b* is negative for blue and positive for yellow. Both a* and b* color values were used to calculate chroma = square root [(a*)^2^ + (b*)^2^] and hue = arctangent (b*/a*) (degrees). Hereafter, only chroma and hue were used within this manuscript. Chroma (saturation or vividness) is reported as chromaticity increases a color becomes more intense and as it decreases a color becomes duller.

### 2.3. Statistical analysis of peach characteristics

Analyses of variance were performed using the ANOVA procedure in SAS software (version 9.2; SAS Institute, Cary, NC) to examine differences between cultivars. Differences among cultivars were examined using Least Significant Difference (LSD) test with a confidence level of 95%.

### 2.4. Multiple linear regression

A multiple linear regression (MLR) model may be written as

$$y=b_{0}+b_{i}X_{i}+\ldots +b_{p}X_{p}$$ (5)

where *b*_0_ is the intercept, y is the output variable, b is the regression coefficient (*i* = 0, 1, 2,…, p) and *X* is the input variable (*i* = 1, 2,…, p). When *b*_0_ and b coefficients are achieved, a mathematical model of prediction can be used to estimate the continuous output as linear functions of independent inputs. The acceptance of regression models may be due to the interpretability of *b*_0_ and b coefficients and simplicity of use. Here, for the prediction of chemical composition parameters of peach fruits, regression analysis was performed using a data analysis tool in Excel software. The selected independent variables were surface color values (L*, Hue and chroma), single fruit weight, juice volume, and sphericity percent. The dependent variables were TSS, titratable acidity, TSS/titratable acidity, vitamin C, anthocyanin and total carotenoids. Thus, six regression equations were created. Regression analyses were performed on the data utilized in the training process of the ANN model and the rest of the data were used to test the performance of the regression models.

### 2.5. Building an artificial neural network model to predict the chemical composition of peach fruits

ANNs use simple processing elements called neurons. They determine the inherent relationship between parameters through learning processes and create a mapping between input space (input layer) and target space (output layer) [31]. A multilayer perceptron network consists of one input layer, one or more hidden layers, and one output layer [32].

The ANN configuration used in this investigation consisted of three layers connected by weights. The weights, denoted by $w_{ji}^{h}$, connect input neuron *i* to hidden neuron *j*. The weights denoted by $w_{j}^{o}$ connect hidden neuron *j* to output neuron. The input of each neuron is the weighted sum of the network inputs. Based on the values of the inputs, the output is processed by a sigmoid transfer function. More specially, for the *j*^th^ hidden neuron [33].


$$\left\{ \begin{array}{l} net_{j}^{h}=\sum_{i=1}^{n}w_{ji}^{h}x_{t-1}+b_{j},\\ y_{j}=f(net_{j}^{h}) \end{array}\right.$$ (6)

While for the output neuron

$$\left\{ \begin{array}{l} net_{}^{o}=\sum_{j=1}^{m}w_{j}^{o}y_{j}+c,\\ {\tilde{x}}_{t}=f(net_{}^{o}) \end{array}\right.$$ (7)

Where *c* and *b*_*j*_ are thresholds (bias). However, in the input layer in the established network, *n* neuro-ns are created, and in the hidden layer, *m* neurons are seen, *f* is usually reserved to be a sigmoidal function, such as the logistic function

$$f(x)=\frac{1}{1+e^{-x}}$$ (8)

The inputs to the network were single fruit weight, juice volume, sphericity percent of fresh peach fruit, and L*, Hue, and chroma. The outputs were TSS, titratable acidity, TSS/ titratable acidity, vitamin C, anthocyanin, and total carotenoids. Given a finite number of pattern pairs consisting of an input pattern *x*_*t*_ and a target output pattern ${\tilde{x}}_{t}$, this network was trained by supervised learning. Generally, the back-propagation algorithm, which is the most popular learning algorithm, was adopted to perform steepest descent on the total mean squared error (MSE):

$$MSE=\frac{1}{2}\sum_{t=1}^{N}{\left({\tilde{x}}_{t}-x_{t}\right)}^{2}$$ (9)

Where *N* is the total number of pattern pairs.

The input and output data were 180 rows as all cultivars were considered together. However, in ANN modeling, using replicate values instead of mean values helps to evaluate deviation in the ANN model [34]. Commercial neural network software of Qnet software for Windows [35] was used to create the ANN model. The architecture of the ANN in this investigation was a standard back-propagation ANN with three layers, namely an input layer, a hidden layer, and an output layer. Before the training stage, the output and input data were normalized to make the training process more efficient. The output and input values ranged between 0.15 and 0.85. The normalization range was created using Eq 10:

$$T=\frac{\left(V-V_{\min}\right)}{\left(V_{\max}-V_{\min}\right)}\times (0.85-0.15)+0.15$$ (10)

**Fig 3:**
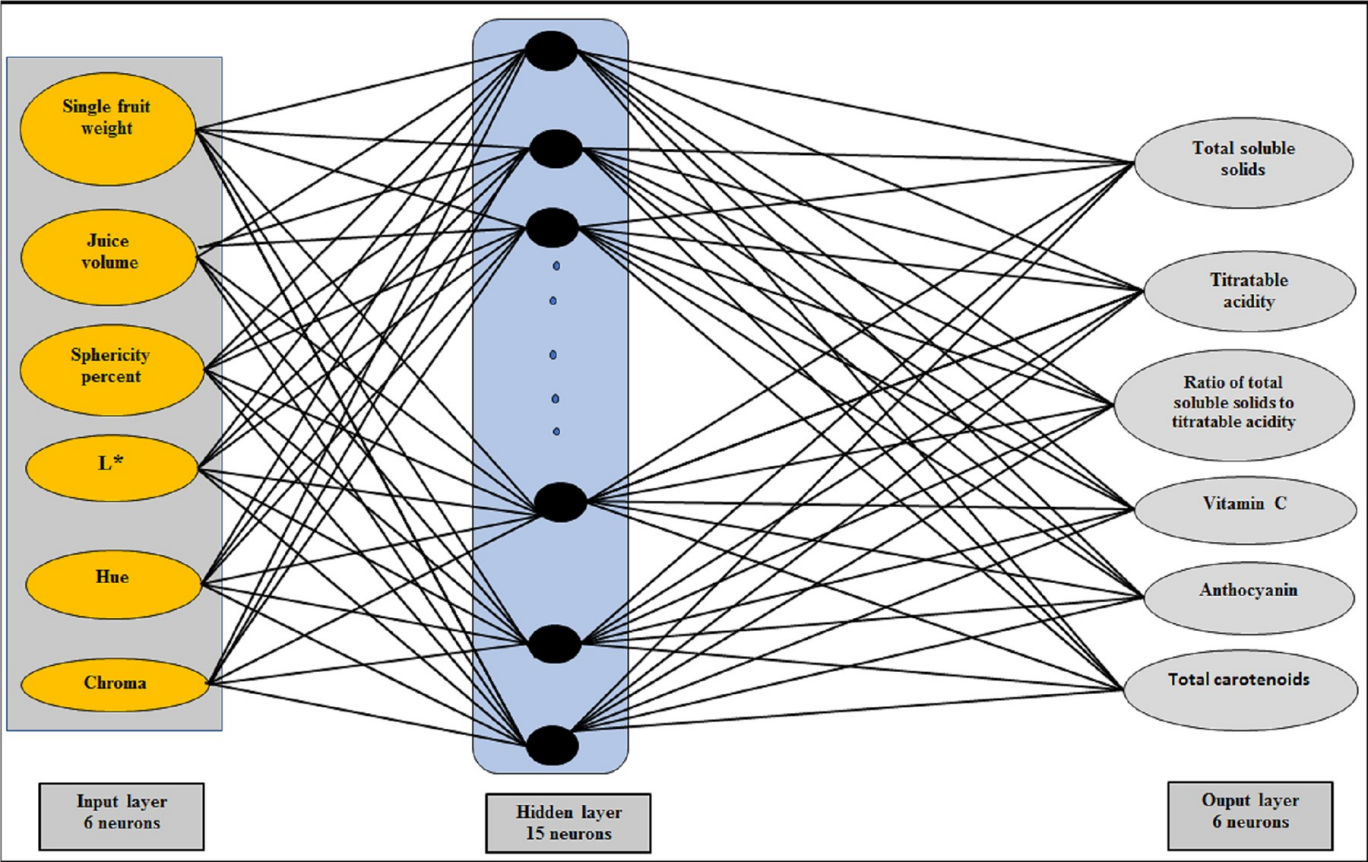
Structure of ANN model to predict total soluble solids, titratable acidity, ratio of total soluble solids to titratable acidity, vitamin C, anthocyanin, and total carotenoids contents of fresh peach fruit.

Where V is the original values of output and input variables, T is the normalized value, and V_min_ and V_max_ are the minimum and maximum values of the output and the input variables, respectively. The software was directed to select 54 points to validate the ANN model, and the rest of the points were used to create the ANN model. A training data set was used to compute all the weights and thresholds of the ANN. Meanwhile, the testing data set was used to evaluate the precision of the ANN model predictions [15]. The number of neurons (n1) in the hidden layer was changed from 1 to 25 to select the best ANN structure. However, the training process was evaluated for a 6-n1-6 ANN structure. Also, different momentum, learning coefficient, and transfer function values were considered. The best ANN structure was obtained on the basis of the lowest error on training data using a trial and error method. The training results indicate that the finest performance to predict the chemical compositions of peach fruit belonged to the 6-15-6 ANN structure (Fig 3). The training parameters as training error was 0.014613, learning rate was 0.034005 and momentum was 0.8 and 100000 for iterations.

### 2.6. Evaluation of the investigated models

The performance of the established ANN and MLR models was evaluated using various statistical criteria. However, the mean absolute error (MAE) and root mean square error (RMSE) were used in addition to coefficient of determination (R^*2*^). The MAE and RMSE were determined according to the following equations:

$$MAE=\frac{1}{N_{a}}\sum_{i=1}^{N_{a}}\left|Y_{a}-Y_{p}\right|$$ (11)

$$RMSE=\sqrt{\frac{\sum_{i=1}^{N_{a}}{\left(Y_{a}-Y_{p}\right)}^{2}}{N_{a}}}$$ (12)

Where Y_a_ and Y_p_ are the observed and predicted data, respectively and *N*_*a*_ is the number of data points in training and testing datasets.

## 3. Results and discussion

### 3.1. Analysis of peach characteristics

Fruit weight, juice volume, sphericity percent, skin color parameters (L*, hue, and chroma), total soluble solids (TSS), titratable acidity, TSS/titratable acidity, vitamin C, anthocyanin, and total carotenoids were evaluated for 9 cultivars of peaches grown in Egypt for the 2019 seasons. Statistical analyses were made on the experimental data to assess the significance of the differences in the peach characteristics of the nine peach cultivars. There were statistical significant differences observed for all parameters (P < 0.0001). Mean separation between cultivars are presented. However, Table 1 depicts mean fruit weight, juice volume, sphericity percent, TSS/titratable acidity and vitamin C evaluated for nine commercial fresh peach cultivars. The smallest juice volume was recorded for the swelling cultivar, with a value of 33.69 ml, and the highest juice volume was recorded in the Desertred cultivar, with a value of 84.12 ml (Table 1). In addition, in (Table 1), the sphericity percent varied between 90.74% and 98.61% and dependent of cultivars. In the study of [24], the ranges of calculated sphericity [(H*W*T)^1/3^/W] (%) of three peach cultivars namely Anjiri, Shali, and Elberta in maturation stage were 79.83–88.80, 98.05–103.55 and 93.54–104.21, respectively.

**Table 1:** Mean fruit weight, juice volume, sphericity percent, TSS/titratable acidity, and Vitamin C for nine commercial peach cultivars.

Peach cultivars	Fruit weight	Juice volume	Sphericity percent	TSS/titratable acidity	Vitamin C
	(g)	(ml)	(%)	(—)	(mg/100ml juice)
Dixon	109.72g	52.89d	90.74f	13.96c	11.42a
Early Grande	120.79f	58.35c	94.74c	11.26g	9.62b
Flordaprince	120.24f	42.62h	98.22a	12.96e	10.63c
Flordastar	109.83g	44.89g	98.61a	12.19f	11.26d
Flordaglo	132.84c	51.95e	93.84e	12.31f	9.66e
Florda 834	127.87e	46.52f	94.51d	13.74d	9.87e
TropicSnow	146.21a	78.77b	93.94de	15.15b	8.39f
Desertred	137.88b	84.12a	96.16b	11.32g	7.93g
Swelling	129.15d	33.69i	98.18a	32.01a	7.77h
LSD	0.58	0.40	0.64	0.24	0.11

Means followed by different letters are significantly different from others (P<0.05).

Fruit weight is a vital feature in quality evaluation [36], and it can impact consumer acceptance, yield, and processing [37]. In addition, it is considered a quantitative factor that defines production, fruit quality and consumer acceptability, as supported in the work of [7], and it is related to the physiology of preach trees [4]. The weights of the investigated peach cultivars varied from 109.72 to 146.21 g (Table 1). In the study of [38], the fruit weight of the Early Grande cultivar in the control treatment was 113.65 g in the 2007 growing season, and it was 111.55 g in the 2008 growing season. In the present study, the fruit weight for the same cultivar was 120.79 g (Table 1). Moreover, for Desertred peach trees, the fruit weight was 82.31 g for the control treatment in the 2016 growing season [39]. In the present study, the weight of Desertred peach fruit was 137.88 g [40] found that the weights of peaches varied from 183.59 to 320.67 g, and [41] reported a weight range of 120 to 275 g for peach fruits. In our investigation, there was more than a 1.33-fold range (109.72 to 146.21 g) in mean fruit weight among the cultivars due to the influence of cultivar and fruit type (flat or round), as indicated by [41]. This agrees with previous studies that found high variability in fruit weight among peaches [42]. However, Williams and Crocker [43] specified that a perfect commercial peach tree must produce a fruit weight in the range of 90 to 150 g.

The use of the TSS/titratable acidity ratio has been proposed as a measure of acceptable flavor quality when kept above a minimum TSS and below a set maximum titratable acidity [44]. In the present study, TSS/titratable acidity ratio was calculated, and values varied between 11.26 and 32.01 (Table 1). The largest TSS/titratable acidity ratio was found for the swelling cultivar and the smallest TSS/titratable acidity ratio was recorded for the Early Grande cultivar. Wahdan and Ismaeil [38] found that the TSS/titratable acidity ratio of the Early Grande cultivar was 12.14 in the 2007 season and 11.00 in the 2008 season for the control treatment., which is similar to our finding of 11.26 for the same cultivar (Table 1), with variation possibly being due to the different areas where it was grown in Egypt. Moreover, for the Desertred peach, the TSS/titratable acidity ratio was 19.18 for the control treatment in the 2016 season [39]. In the present study, the TSS/titratable acidity ratio in the Desertred cultivar was 11.32. Previous studies have linked the sweetness perception of consumers with high TSS/titratable acidity ratio values [9, 11, 45–47], where the cultivar with the highest ratio was considered the sweetest cultivar [9]. In the present study, the swelling cultivar was considered to be the sweetest cultivar.

For vitamin C, the Dixon cultivar showed the highest value11.42 mg/100ml juice, and the lowest value was found for the Early Grande cultivar 9.62 mg/100ml juice. As previously reported for the vitamin C contents in peach flesh, values ranged from 16.70 mg/100 g in the 2007 season to 15.85 mg/100 g in the 2008 season for the control treatment of the Early Grande cultivar [38]. Moreover, for Desertred peach trees, the vitamin C content was 14.72 mg/100ml juice for the control treatment in the 2016 season [39]. In the present study, the TSS/titratable acidity ratio in the Desertred cultivar was 7.93 mg/100 ml juice.

**Fig 4:**
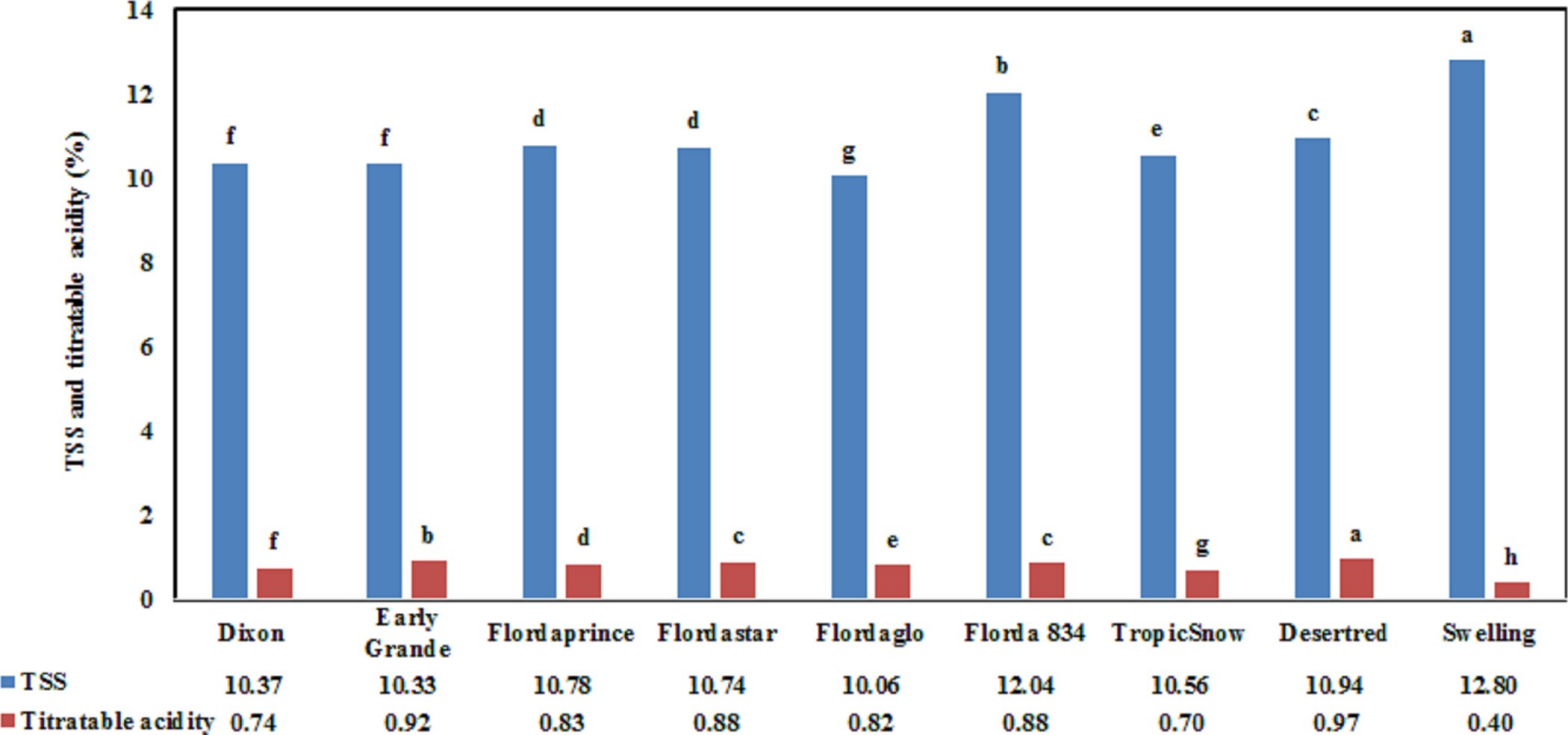
Mean TSS and titratable acidity evaluated for nine commercial fresh peach cultivars.

TSS is used as an indicator of sweetness, and titratable acidity is used as an indicator of sourness [11]. Variability in sugar concentrations has been attributed to the inherent variation among cultivars [48]. TSS is the most important quality parameter used to indicate the sweetness of fresh and processed horticultural food products in laboratories for research and by industry to determine marketing standards [49]. Significant differences among cultivars were identified for TSS and titratable acidity (P < 0.0001). Fig 4 shows mean TSS and titratable acidity evaluated for nine commercial fresh peach cultivars. All tested cultivars exhibited TSS within an acceptable range as the lower value was 10.06%, which was observed for the Flordaglo cultivar, and the highest TSS was 12.80%, which was observed for the swelling cultivar. Wahdan and Ismaeil [38] found that the TSS of the Early Grande cultivar for the control treatment was 8.5% in the 2007 growing season and 7.7% in the 2008 growing season. However, in the present study, the fruit TSS for the same cultivar was 10.33% (Fig 4), and this variation may be due to the different areas that the cultivar was grown in. Moreover, for Desertred peach trees, the TSS was 11.7% for the control treatment in the 2016 growing season [39]. The TSS in the Desertred cultivar was 10.94%, which is slightly lower than the findings of [41], who reported TSS in the range of 11.60%–16.40%. Several studies have associated high consumer acceptance with high TSS in peaches [50, 51]. The quality standards for yellow-flesh peaches in California have been set at a minimum of 10% TSS [52]. A TSS value below 10% is generally unacceptable to consumers [53].

Titratable acidity varied between 0.40% and 0.97% (Fig 4). The highest value for titratable acidity was found for the Desertred cultivar, and the lowest titratable acidity was observed for the swelling cultivar, as the variation in fruit acidity was associated with cultivar type. Wahdan and Ismaeil [38] found that the titratable acidity of the Early Grande cultivar was 0.7% in the 2007 season and 0.7% in the 2008 season for the control treatment. In the present study, the fruit titratable acidity was 0.92% for the same cultivar (Fig 4). This difference could be due to where this cultivar was grown in Egypt. Moreover, for the Desertred peach trees, the titratable acidity was 0.6% for the control treatment in the 2016 growing season [39]. In the present study, the titratable acidity in the Desertred cultivar was 0.97%. However, Fathi *et al*. [41] reported titratable acidity of 0.54% % to 0.92% for their peach cultivars. Also, the present values for TSS are similar to those reported by [54] with a range from 10.9% to 13.8% and [55] with a range of 7.6% to 17.5%.

**Fig 5:**
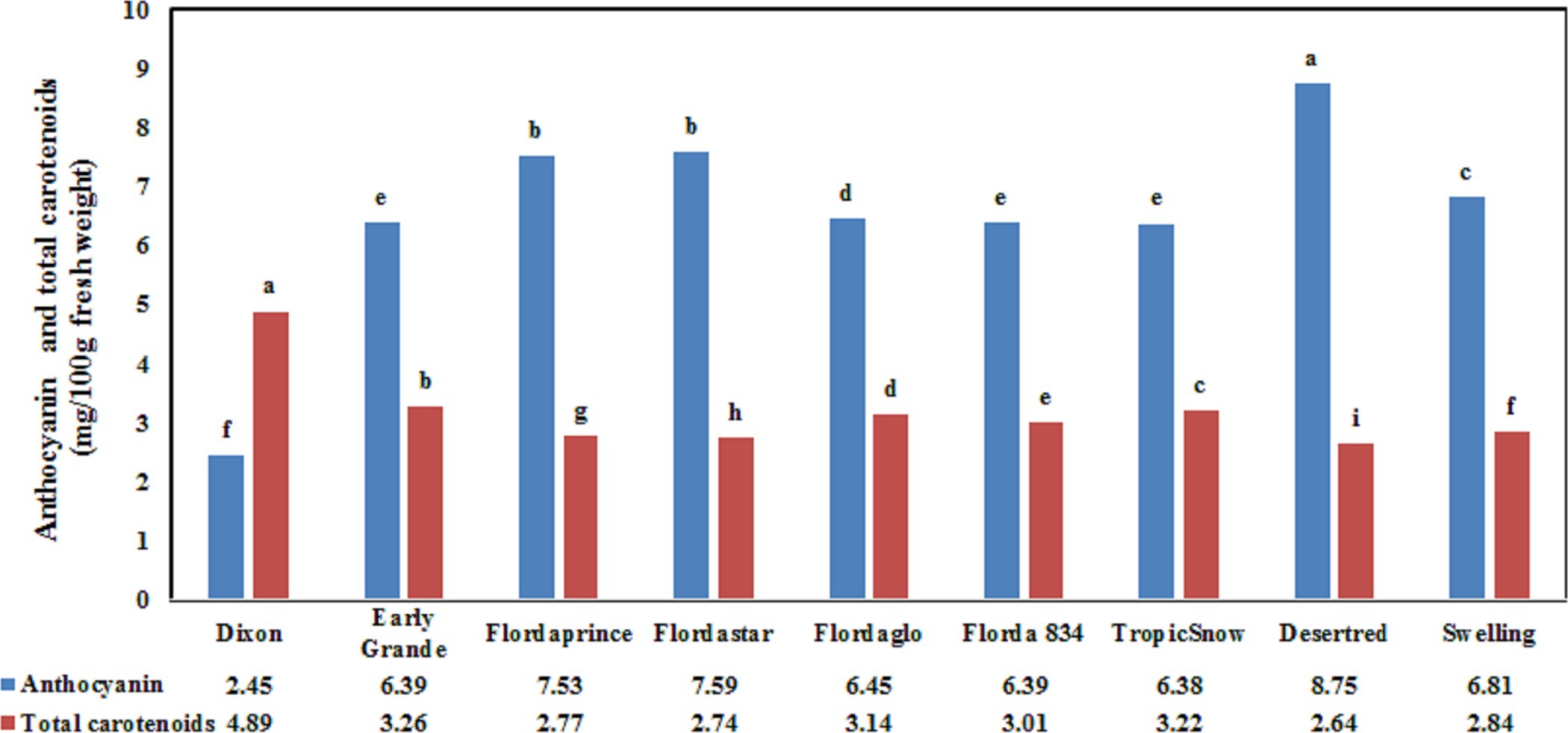
Mean anthocyanin and total carotenoids evaluated for nine commercial fresh peach cultivars.

There were statistical significant differences observed for anthocyanin content and total carotenoids (P < 0.0001) when comparing cultivars. For anthocyanin content, the Desertred cultivar induced the highest values of 8.75 mg/100 g fresh weight, and the lowest value was induced by Dixon (2.45 mg/100 g of fresh weight) as indicated in Fig 5. However, Abd El-Megeed and Medan [39] reported an anthocyanin value in the Desertred cultivar of 13.61 mg/100 g of fresh weight.

Carotenoids play an important role in human health by acting as sources of vitamin A or as protective antioxidants [56]. In this study, for the evaluation of total carotenoids, the Dixon cultivar induced the highest values of 4.89 mg/100 g of fresh weight, and the lowest value was induced by the Desertred cultivar (2.64 mg/100 g fresh weight). However, Abd El-Megeed and Medan [39] reported a carotene value in the Desertred cultivar of 6.37 mg/100 g of fresh weight.

**Fig 6:**
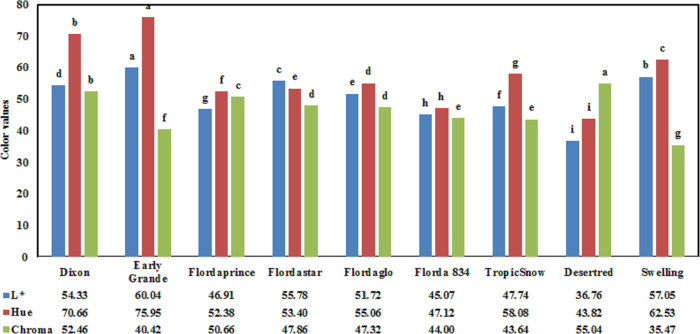
Mean L*, Hue, and chroma for surface skin evaluated for nine commercial fresh peach cultivars.

Skin color was evaluated for peaches in the 2019 season. There were statistical significant differences across for L*, hue and chroma (P < 0.0001), hence mean comparisons are presented (Fig 6). By observing Fig 6, L* varied from 36.76 to 60.04, and the Early Grande cultivar had the highest average lightness (L* = 60.04). In the study of [57], L* varied between 80.32 and 91.72 for commercial peach cultivars of Greta, UFO4, Rome Star, and UFO6. Additionally, in the study of Belisle et al. [11], skin L* values ranged from 32.89 for ‘Springflame’ to 76.06 for ‘Julyprince’ in 2015 and from 31.20 for ‘Springflame’ to 73.49 for ‘Augustprince’ in 2016 for fresh peach cultivars. For hue, values for skin ranged 43.82 to 75.95 (Fig 6). However, Hue (tint of color) is an angular measurement where 0 = red, 45 = orange-red, 90 = yellow, 180 = green, and 270 = blue [11]. However, in the study of Belisle et al. [11], for hue values for some peach cultivars, the range was 29.31 for ‘Springflame’ to 78.20 for ‘Elberta’ in 2015, and from 29.37 for ‘White Lady’ to 74.54 for ‘Elberta’ in 2016. In chroma, values for skin ranged from 35.47 to 55.04. In the study of Belisle et al. [11], for chroma values for some peach cultivars, the range was 30.56 for ‘Springflame’ to 63.01 for ‘Ruston Red’ in 2015 and from 31.58 for ‘Rich Lady’ to 57.62 for ‘Augustprince’ in 2016. Previous studies also reported that fruit color can vary from year to year depending on sunlight and temperature. In addition, temperature has a major influence on anthocyanin synthesis for coloring in fruit species [58]. The primary pigments imparting color quality are the fat-soluble chlorophylls (green), carotenoids (yellow, orange, and red), water-soluble anthocyanin (red, blue), flavonoids (yellow), and betalains (red) [59].

### 3.2. Analysis of multiple linear regression models

In this study, six regression formulas were established to predict TSS, titratable acidity, TSS/titratable acidity, vitamin C, anthocyanin, and total carotenoids of peach from easily measurable parameters of surface color values (L*, Hue and chroma), single fruit weight, juice volume, and sphericity percent. Table 2 shows regression coefficients of different chemical compositions based on Eq (5) with coefficients of determination (R^2^) using training data set. According to the results of the regression coefficients (Table 3), L*, Hue, chroma, single fruit weight, juice volume, and sphericity percent were found to differently affect the investigated chemical attributes. The amounts of these factors showed various relationships with the content of chemical attributes, as well.

**Table 2:** Regression coefficients of different chemical compositions based on Eq (5) with coefficients of determination (R^2^) using training data set.

Variables	TSS	Titratable acidity	TSS/titratable acidity	Vitamin C	Anthocyanin	Total carotenoids
	(%)	(%)	(—)	(mg/100ml juice)	(mg/100g fresh weight)	(mg/100g fresh weight)
Intercept	18.381	2.426	-44.226	23.368	-31.694	14.366
Fruit weight (g)	-0.006	-0.012	0.340	-0.072	0.005	-0.002
Juice volume (ml)	-0.022	0.009	-0.301	-0.006	0.046	-0.011
Sphericity (%)	0.052	0.000	0.373	-0.090	0.411	-0.139
L* (—)	-0.091	-0.002	-0.125	0.047	0.038	-0.014
Hue (—)	-0.003	-0.006	0.212	-0.028	-0.075	0.036
Chroma (—)	-0.120	-0.002	-0.192	0.075	-0.039	0.033
R^2^	0.619	0.554	0.598	0.722	0.812	0.821

**Table 3:** Error criteria for the prediction of chemical composition parameters in peach fruit using multiple linear regression and artificial neural network models for training data sets.

Parameters	Multiple linear regression			Artificial neural network		
	R^2^	MAE	RMSE	R^2^	MAE	RMSE
TSS (%)	0.619	0.456	0.570	0.987	0.078	0.105
Titratable acidity (%)	0.544	0.095	0.109	0.944	0.009	0.013
TSS/titratable acidity (—)	0.598	3.456	0.484	0.994	0.326	0.484
Vitamin C (mg/100ml juice)	0.722	0.558	0.680	0.989	0.105	0.138
Anthocyanin mg/100g fresh weight)	0.812	0.532	0.672	0.994	0.093	0.124
Total carotenoids (mg/100g fresh weight)	0.821	0.204	0.257	0.998	0.022	0.029

### 3.3. Analysis of artificial neural network modeling

We used the measured data to verify the capability of the ANN to predict chemical composition. We used surface color values (L*, hue and chroma), single fruit weight, juice volume, and sphericity percent as input variables. However, TSS, titratable acidity, TSS/titratable acidity, vitamin C, anthocyanin, and total carotenoids were acted the output variables of the established ANN model. The value of every chemical composition was predicted according to the trained network in one model. The predicted and experimental datasets of training samples were compared and the results presented in Table 3 show the high ability of the ANN to produce outputs similar to the experimental data set. A high correlation between the predicted results and outputs is evident. The testing accuracy of *R*^2^ was in the range of 0.987 to 0.998, which outperformed the multiple regression models (R^*2*^ = 0.554–0.821).

**Fig 7:**
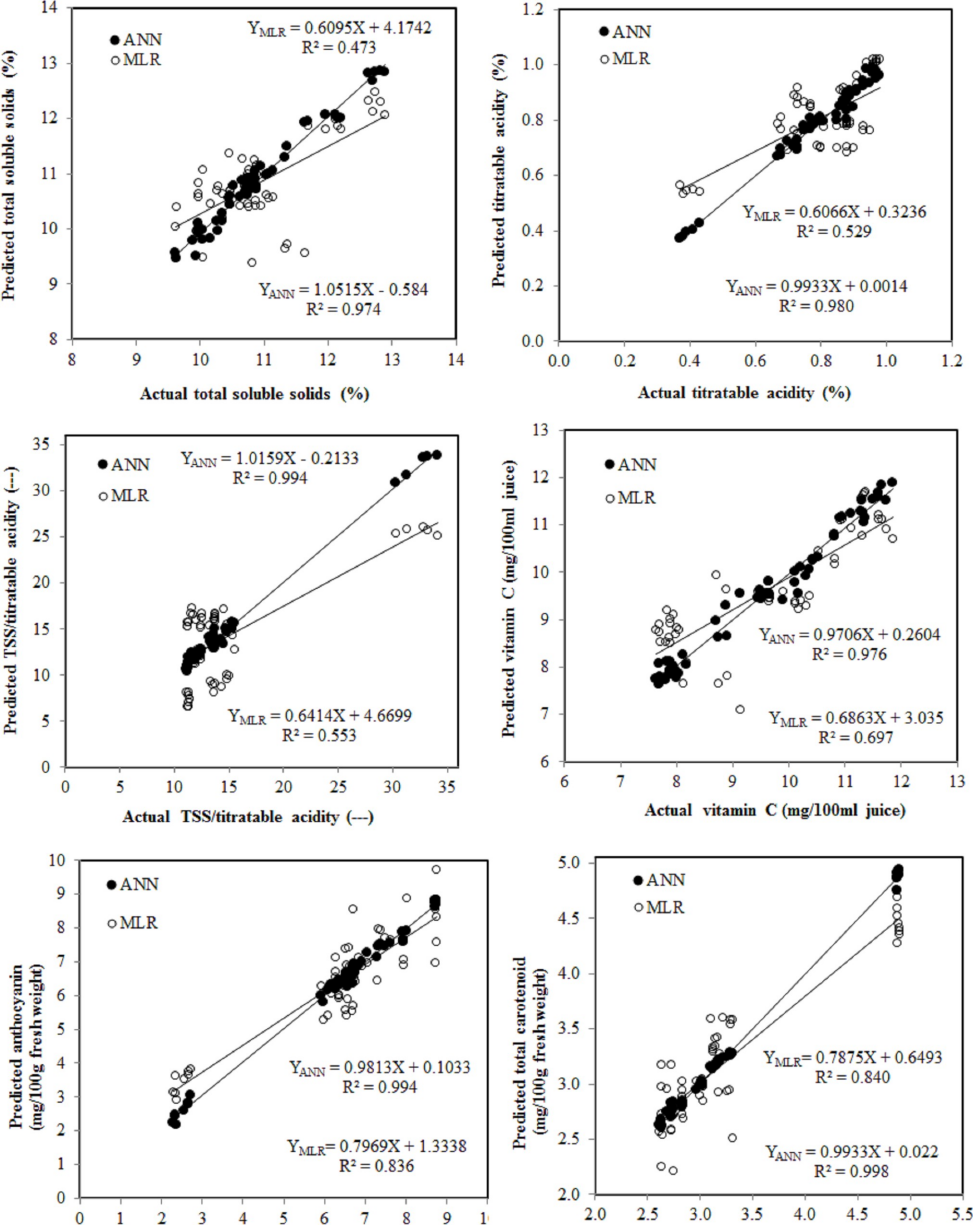
Regression analysis between the desired output and the obtained output variables.

The predicted and experimental datasets of testing samples were also compared to test the performance of the developed ANN model, and the results presented in Table 4 show the high ability of the ANN to produce outputs similar to the experimental data using testing data set. The testing accuracy of *R*^2^ was in the range of 0.974 to 0.998, which outperformed the multiple regression models (R^*2*^ = 0.473–0.840), indicating that the established ANN model is efficient and feasible. The R^2^ statistics for our developed ANN model are highly constant for both the training and test data predictions of each output (Tables 3 and 4), suggesting a lack of over fitting throughout the testing process and this is seen in Fig 7. However, ANN model validation, Fig 7 describes the relationship between the desired output and obtained output variables. It is observed that the value of R^2^ is 0.974 for TSS, showing that approximately 97.4% of the observed TSS variable can be explained concerning desired TSS, that is, more homogeneous are the data with a strongly positive linear correlation [60] and so on for other variables. The estimated statistical error criteria and R^2^ related to the ANN model revealed a substantially higher accuracy of prediction than for the MLR models (Tables 3 and 4). Therefore, the use of the sigmoid activation function provides a rational choice for modeling non-linearities over all data. The strength of our study is that we used the same datasets (training and testing) for developing the ANN model and MLR, which confirms that the developed models are quite reliable and valid.

**Table 4:** Error criteria for the prediction of chemical composition parameters in peach fruit using multiple linear regression and artificial neural network models for testing data sets.

Parameters	Multiple linear regression			Artificial neural network		
	R2	MAE	RMSE	R2	MAE	RMSE
TSS (%)	0.473	0.467	0.632	0.974	0.121	0.151
Titratable acidity (%)	0.529	0.093	0.108	0.980	0.016	0.022
TSS/titratable acidity (—)	0.553	3.511	3.993	0.994	0.384	0.483
Vitamin C (mg/100ml juice)	0.697	0.627	0.762	0.976	0.171	0.218
Anthocyanin mg/100g fresh weight)	0.836	0.613	0.750	0.994	0.111	0.142
Total carotenoids (mg/100g fresh weight)	0.840	0.229	0.295	0.998	0.025	0.035

The estimated statistical values using testing dataset related to the ANN model revealed a substantially higher accuracy of prediction than for regression models, so as calculated R^2^ for ANN vs. regression models were: TSS = 0.974 vs. 0.473, Titratable acidity = 0.980 vs. 0.529, TSS/titratable acidity = 0.994 vs. 0.553, Vitamin C = 0.976 vs. 0.697, Anthocyanin = 0.994 vs. 0.836, total carotenoids = 0.998 vs. 0.840 (Table 4). In order to build an accurate prediction model, it is essential to use a dependable modeling method to predict subjects [15].

The ANN model is recommended as a promising prediction tool to determine the chemical compositions of TSS, titratable acidity, TSS/ titratable acidity, vitamin C, anthocyanin, and total carotenoids in peach cultivars based on easily measurable parameters of single fruit weight, juice volume, sphericity percent, L*, hue, and chroma. The established technique also provides a new potential approach for peach quality inspection research into other fruits. Also, such mathematical relationships and predictions using MLR have not been previously reported by researchers in this area. ANN-based models were compared with MLR models considering the accuracy of the prediction. ANN are able to learn difficult relationships and simplify results from given patterns of input/output data. Therefore, ANN is a suitable method for the modeling of complicated problems for which precise models or feasible performances have not been developed. Solving a problem using ANNs can be influenced by the quality of the training data, data type, and architecture of the ANN and the learning algorithm for that specified case [61]. The key advantage of an ANN model is that it is not necessary to specify a previous closely fitting function: it has an ability to predict different types of non-linear functions, supporting the creation of the most precise prediction model. Based on the high level of accuracy of the predicted data in both the training and testing stages, we suggest that the established ANN model is adept for predicting the chemical content of peaches. Previous prediction studies in different research fields have also shown considerably higher precision of ANN modeling methods than regression modeling [62]. Finally, predicting the quantity of chemical compositions in peach fruits as enhanced raw material for industrial or research applications is highly helpful as knowledge of the chemical composition profiles and quality attributes of the peach samples will assist with selecting the most suitable peach samples during the harvesting period and increasing the price of potential commercial peaches.

### 3.4. Contribution analysis

In order to determine the relative importance of input variables, the entire dataset was used to estimate the contribution percentage for each input, which indicates how the change in each input affects the output prediction. The contribution percentage of the six input variables to the outputs was calculated using the established ANN model, and the results are illustrated in Table 5 for TSS, titratable acidity, TSS/ titratable acidity, vitamin C, anthocyanin, and total carotenoids in peach cultivars. The higher the contribution percentage value, the more important is the input variable. Thus, the inputs can be ranked according to their importance in determining the outputs using the contribution percentage values.

Among the input variables, hue on TSS prediction was the highest compared with the other parameters, with a contribution percentage of 20.86%. According to the obtained contribution percentage values, the juice volume had the highest contributions to titratable acidity, TSS/titratable acidity and vitamin C with corresponding contribution values of 39.97%, 50.40%, and 33.08%, respectively. Sphericity percent contributed by 65.24% to titratable acidity. In addition, sphericity percent contributed by 23.70% to anthocyanin and by 24.08% to total carotenoids. Hue angle contributed by 77.85% to anthocyanin. Chroma contributed by different values to all variables in the range of 5.29% to 19.39% (Table 5). Moreover, L* contributed by different values to all variables in the range of 6.17% to 9.94% (Table 5). Furthermore, fruit weight contributed by different values to all variables in the range of 16.67% to 23.48% (Table 5). Accordingly, pomology engineers can establish and pay attention to the parameters that dominate the state of the output and ignore those having small contributions.

**Table 5:** Contribution percentage of six independent variables used in the artificial neural network model for predicting TSS, titratable acidity, TSS/titratable acidity, vitamin C, anthocyanin, and total carotenoids in peach cultivars.

Input node	Percent contribution (%)					
	TSS	Titratable acidity	TSS/titratable acidity	Vitamin C	Anthocyanin	Total carotenoids
	(%)	(%)	(—)	(mg/100ml juice)	(mg/100g fresh weight)	(mg/100g fresh weight)
Fruit weight	19.61	23.48	20.48	20.32	18.04	16.67
Juice volume	18.85	39.97	50.40	33.08	20.96	10.94
Sphericity percent	15.03	10.96	7.23	11.52	23.70	24.08
L*	8.60	9.20	9.94	7.48	7.78	6.17
Hue	20.86	10.24	6.65	17.77	16.46	22.76
Chroma	17.05	6.15	5.29	9.83	13.06	19.39

## 4. Conclusions

Descriptive statistics of quality attributes of nine peach cultivars were considered and provide basic information for further investigation in different peaches cultivars. In the current study, using color values (L*, Hue and chroma), single fruit weight, juice volume, and sphericity percent of fresh peach fruit as input variables, the ANN and MLR method were used to predict TSS, titratable acidity, TSS/titratable acidity, and vitamin C, anthocyanin, and total carotenoids levels in peach fruits. The results showed that the ANN model compared to the MLR model could predict the TSS, titratable acidity, TSS/titratable acidity, and vitamin C, anthocyanin, and total carotenoids with more accuracy (R^2^ = 0.974, R^2^ = 0.980, R^2^ = 0.994, R^2^ = 0.976, R^2^ = 0.994 and R^2^ = 0.998, respectively) in testing stage. According to sensitivity tests, the Hue of fruit skin is the most important independent variable predicting TSS in the ANN model, the juice volume in fruit is the most important independent variable predicting titratable acidity, TSS/titratable acidity, and vitamin C in the ANN model, and the sphericity percent is the most important independent variable to predict anthocyanin and total carotenoids in ANN model. The ANN model established in this study can clearly know which input variables are relatively important for fruit quality. In actual production, each regression factor (TSS, titratable acidity, TSS/titratable acidity, vitamin C, anthocyanin, and total carotenoids) can be optimized by adjusting the investigated independent variables to improve the quality of fresh peach fruit. Furthermore, the acquired results are promising from the standpoint that pomology engineers can determine the chemical compositions of peach fruits at harvesting time.

## Supporting information

S1 Data (XLSX)

## References

[1] Rakonjac V, Živanović T. Stability of yield and fruit quality in promising peach cultivars. Journal of Central European Agriculture. 2008; 9 (1): 177–184.

[2] Khalifa SM, Hamdy A E. Effect of some pre-harvest treatments on yield and fruit quality of "Swelling" peach trees. Annals of Agricultural Science, Moshtohor. 2018; 56 (2): 397–404.

[3] Alexandre HV, de Figueirêdo RMF, de Melo Queiroz AJ, de Oliveira ENA. 2014. Storage of surinam cherry powder. Comunicata Scientiae. 2014; 5 (1): 83–91 (Abstract in English).

[4] García EQ, Casierra-Posada F, Contreras A E D. Quality of peach fruits Jarillo cv. (*Prunus persica* L.) in Pamplona, Colombia. Revista Brasileira de Fruticultura. 2018; 40 (6), 1–14. http://dx.doi.org /10.1590/0100-29452018040

[5] Morais KD B, Xavier B, Da S, Silva DP, Oliveira JA A. Bruckner, C.H. Physical and chemical evaluation of sixteen peach cultivars during three harvests. Engenharia na Agricultura. 2017; 25(2):157–163 (Abstract in English)

[6] Matias RGP, Silva DFP, Miranda PMD, Oliveira JAA. Pimentel, L.D.; Bruckner, C.H. Relationship between fruit traits and contents of ascorbic acid and carotenoids in peach. Crop Breeding and Applied Biotechnology. 2016; 16 (4): 348–354. 10.1590/1984-70332016v16n4n51

[7] Matias RGP, Bruckner CH, Silva DFP, Carneiro PCS, Oliveira JAA. Adaptability and stability of peach and nectarine cultivars in subtropical climate. Revista Ceres. 2017; 64 (5): 516–522. 10.1590/0034-737x201764050009

[8] Silva DFP, Matias RGP, Costa E, Silva JO, Salazar AH, Bruckner CH. Characterization of white-fleshed peach cultivars grown in the ‘Zona da Mata’ area of Minas Gerais State, Brazil. Comunicata Scientiae. 2016; 7(1): 149–153. doi: 10.14295/cs.v7i1.781

[9] Colaric M, Veberic R, Stampar F, Hudina M. Evaluation of peach and nectarine fruit quality and correlations between sensory and chemical attributes. Journal of the Science of Food and Agriculture.2005; 85: 2611–2616. doi: 10.1002/jsfa.2316

[10] Kader AA. Flavor quality of fruits and vegetables. J. Sci. Food Agric. 2008; 88: 1863–1868.

[11] Belisle C, Phan UTX, Adhikari K, Chavez DJ. A fruit quality survey of peach cultivars grown in the Southeastern United States. HortTechnology. 2018; 28 (2): 189–201. 10.21273/HORTTECH03870-17

[12] Harker FR, Marsh KB, Young H, Murray SH, Gunson FA, Walker SB. Sensory interpretation of instrumental measurements 2: sweet and acid taste of apple fruit. Postharvest Biology and Technology. 2002; 24: 241– 250.

[13] AOAC. Vitamins and other nutrients (Chapter 45). In *Official Methods of Analysis* 17th ed., D.C. Gaithersburg Maryl. 2005; U.S. A.

[14] Khalifa S, Komarizadeh MH, Tousi B. Usage of fruit response to both force and forced vibration applied to assess fruit firmness- a review. Austrailain Journal of Crop Science. 2011; 5 (5):516–522.

[15] Eftekhari M, Yadollahi A, Ahmadi H, Shojaeiyan A, Ayyari M. Development of an artificial neural network as a tool for predicting the targeted phenolic profile of grapevine (*Vitis vinifera*) foliar wastes. Frontiers in plant Science. 2018; 9, 837. doi: 10.3389/fpls.2018.00837 29971086 PMC6018394

[16] Khairunniza-Bejo S, Mustaffha S, Ismail WIW. Application of artificial neural network in predicting crop yield: A review. Journal of Food Science and Engineering. 2014; 4: 1–9.

[17] Bertolaccini L, Solli P, Pardolesi A, Pasini A. An overview of the use of artificial neural networks in lung cancer research. Journal of Thoracic Disease. 2017; 9 (4): 924–931. doi: 10.21037/jtd.2017.03.157 28523139 PMC5418299

[18] Furferi R, Carfagni M, Daou M. Artificial neural network software for real time estimation of olive oil qualitative parameters during continuous extraction. Computers and Electronics in Agriculture. 2007; 55: 115–131. 10.1016/j.compag.2006.12.006

[19] Rai P, Majumdar GC, DasGupta S, De S. Prediction of the viscosity of clarified fruit juice using artificial neural network: A combined effect of concentration and temperature. Journal of Food Engineering. 2005; 68: 527–533. 10.1016/j.jfoodeng.2004.07.003

[20] Yalcin H, Toker OS, Ozturk I, Dogan M, Kism O. Prediction of fatty acid composition of vegetable oils based on rheological measurements using nonlinear models. European Journal of Lipid Science and Technology. 2012; 114: 1217–1224. 10.1002/ejlt.201200040

[21] Cimpoiu C, Cristea V, Hosu A, Sandru M, Seserman L. Antioxidant activity prediction and classification of some teas using artificial neural networks. Food chemistry.2011; 127: 1323–1328. doi: 10.1016/j.foodchem.2011.01.091 25214133

[22] Huang X, Wang H, Luo W, Xue S, Hayat F, Gao Z. Prediction of loquat soluble solids and titratable acid content using fruit mineral elements by artificial neural network and multiple linear regression. Scientia Horticulture. 2021; 278: 109873. 10.1016/j.scienta.2020.109873

[23] Suárez MH, Dopazo GA, López DL. Espinosa F. Identification of relevant phytochemical constituents for characterization and authentication of tomatoes by general linear model linked to automatic interaction detection (GLM-AID) and artificial neural network models (ANNs). PLoS One. 2015; 10: e0128566. 10.1371/journal.pone.0128566 doi: 10.1371/journal.pone.0128566 26075889 PMC4467870

[24] Zohrabi S, Seiiedlou S, Alipasandi A. Study some physical and mechanical properties of three cultivars of peach in Maturation Stages. World of Sciences Journal. 2013; 04:108–117.

[25] Vivek K, Mishra S, Pradhan RC. Physicochemical characterization and mass modelling of Sohiong (*Prunus nepalensis* L.) fruit. Journal of Food Measurement and Characterization. 2018; 12:923–936. 10.1007/s11694-017-9708-x

[26] Rabino L, Alberto L, Monrad M.K. Photocntrol of anthocyanin synthesis. Journal of Plant physiology. 1977; 59: 569–573 doi: 10.1104/pp.59.4.569 16659895 PMC542450

[27] Moran R, Porath D. Carotenoids determination in intact tissues. Plant Physiol. 1980; 65: 479. 10.1104/pp.65.3.478 PMC440358 16661217

[28] Yam KL, Papadakis SE. A simple digital imaging method for measuring and analyzing color of food surfaces. Journal of Food Engineering. 2004; 61: 137–142.

[29] Adobe Systems. Adobe Photoshop 7.0. User Guide. San Jose., CA: Adobe Systems Inc. 2002.

[30] Kortei NK, Odamtten GT, Obodai M, Appiah V, Akonor PT. Determination of color parameters of gamma irradiated fresh and dried mushrooms during storage. Croatian Journal of Food Technology, Biotechnology and Nutrition. 2015; 10 (1–2), 66–71.

[31] Chayjan RA, Montazer GA, Hashjin TT, Khoshtaghaza MH, Ghobadian B. Prediction of pistachio thermal conductivity using artificial neural network approach. International Journal of Agriculture & Biology. 2007; 9 (6): 816–820.

[32] Hassan-Beygi SR, Ghobadian B, Kianmehr MH, Chayjan R.A. Prediction of a power tiller sound pressure levels in octave frequency bands using artificial neural networks. International Journal of Agriculture & Biology. 2007; 9 (3), 494–498.

[33] Zhang D, Jiang Q, Li X. Application of neural networks in financial data mining. International Journal of Computational Intelligence. 2005; 1 (2):106–109.

[34] Silva SF, Rodrigues Anjos CA, Cavalcanti RN, Celeghini RM. Evaluation of extra virgin olive oil stability by artificial neural network. Food Chemistry. 2015; 179: 35–43. doi: 10.1016/j.foodchem.2015.01.100 25722136

[35] Vesta Services, Inc., 2000. Qnet2000 Shareware. Vesta Services, Inc., 1001 Green Bay Rd, STE 196, Winnetka, IL 60093.

[36] Marini RP, Sowers DL. Peach fruit weight is influenced by crop density and fruiting shoot length but not position on the shoot. Journal of the American Society for Horticultural Science. 1994; 119 (2): 180–184.

[37] Sansavini S, Corelli-Grappadelli L. Yield and light efficiency for high quality fruit in apple and peach high density planting. ISHS Acta Horticulturae.1996; 451:559–568.

[38] Wahdan MT, Ismaeil FHM. Influence of choline chloride on quality and storability of peach fruits cv. Earligrande. Journal of American Science. 2011; 7 (7): 373–381. doi: 10.7537/marsjas070111.50

[39] Abd El-Megeed N A, Medan RA. Effect of foliar application boron and calcium on yield and fruit quality of ’Desert Red" peach trees. Tikrit Journal for Agricultural Sciences. 17, Special 6th Scientific Conference for Agricultural Researches. 2017; 28–29 March, 1–8.

[40] Lyu J, Liu X, Bi J, Jiao Y, Wu X, Ruan W. Characterization of Chinese white-flesh peach cultivars based on principle component and cluster analysis. Journal of food science and Technology. 2017; 54 (12): 3818–3826. doi: 10.1007/s13197-017-2788-0 29085124 PMC5643796

[41] Fathi H, Dejampour J, Jahani U, Zarrinbal M. Tree and fruit characterization of peach genotypes grown under Ardabil and East Azarbaijan environmental conditions in Iran. Crop Breeding Journal. 2013; 3 (1): 31–43.

[42] Quilot B, Kervella J, Génard M. Shape, mass and dry matter content of peaches of varieties with different domestication levels. Scientia Horticulturae. 2004; 99, 387–393.

[43] Williams JG, Crocker T.E. Peaches and nectarines for Florida University of Florida Extension Institute of Food and Agricultural Science, Gainesville, Florida, CIR 299D. 2000; pp.10

[44] Kader A.A. *Postharvest technology of horticultural crops*. 3^rd^ ed. 2002; Univ. California ANR Publ. 3311.

[45] Bassi D, Selli R. Evaluation of fruit quality in peach and apricot. Advances in Horticulture Science.1990; 4:107–112.

[46] Lopez G, Behboudian MH, Echeverria G, Mata M, Girona J, Marsal J. Instrumental and sensory evaluation of fruit quality for ‘Ryan’s Sun’ peach grown under deficit irrigation. HortTechnology. 2011; 21:712–719.

[47] Ortiz A, Lara I, Graell J, Lopez ML, Echeverria G.Sensory acceptance of CA-stored peach fruit. Relationship to instrumental quality parameters. ISHS Acta Horticulturae.2008; 796: 225–230.

[48] Cirilli M, Bassi D, Ciacciulli A. Sugars in peach fruit: A breeding perspective. Horticulture Research. 2016; 3: 15067; doi: 10.1038/hortres.2015.67 26816618 PMC4720000

[49] Magwaza LS, Opara UL. Analytical methods for determination of sugars and sweetness of horticultural products: a review. Scientia Horticulturae. 2015; 184: 179–192. 10.1016/j.scienta. 2015.01.001

[50] Parker D, Ziberman D. Moulton K. How quality relates to price in California fresh peaches. California Agriculture.1991; 45: 14–16.

[51] Crisosto CH, Crisosto GM. Relationship between ripe soluble solids concentration (RSSC) and consumer acceptance of high and low acid melting flesh peach and nectarine (*Prunus persica* (L.) Batsch) cultivars. Postharvest Biology and Technology. 2005; 38: 239–246. doi: 10.1016/j.postharvbio.2005.07.007

[52] Kader AA. *Fruit maturity*, *ripening*, *and quality relationships*. Perishables Handling Newsl. 1995; 80, 2.

[53] Clareton, M. Peach and nectarine production in France: Trends, consumption and perspectives. In *Summaries of the Prunus Breeders Meeting*; Embrapa Cliama Temperado: Pelotas, Brazil. 2000; 83–91.

[54] Contador L, Rubio P, Shinya P, Menese C, Pena-Neira A, Infante R. Phenolics contents and sensory characterization of melting and non-melting peach. The Journal of Horticultural Science and Biotechnology. 2011; 86:255–260.

[55] Cantín CM, Gogorcena Y, Moreno MA. Analysis of phenotypic variation of sugar profile in different peach and nectarine [*Prunus persica* (L.) Batsch] breeding progenies. Journal of the Science of Food and Agriculture. 2009; 89: 1909–1917.

[56] Itle RA, Kabelka EA. Correlation between lab color space values and carotenoids content in pumpkins and squash (Cucurbita spp.). HortScience. 2009; 44 (3):633–637

[57] Di Vaio C, Marallo N, Graziani G, Ritienib A, Di Matteoa A. Evaluation of fruit quality, bioactive compounds and total antioxidant activity of flat peach cultivars. Journal of the Science of Food and Agriculture. 2015; 95 (10): 2124–2131. doi: 10.1002/jsfa.6929 25257768

[58] Mori K, Goto-Yamamoto N, Kitayama M, Hashizume K. Loss of anthocyanins in red-wine grape under high temperature. Journal of Experimental Botany. 2007; 58 (8):1935–1945. doi: 10.1093/jxb/erm055 17452755

[59] Barrett DM, Beaulieu JC, Shewfelt R. Color, flavor, texture, and nutritional quality of fresh-cut fruits and vegetables: desirable levels, instrumental and sensory measurement, and the effects of processing. Critical Reviews in Food Science and Nutrition. 2010; 50 (5):369–389 doi: 10.1080/10408391003626322 20373184

[60] Pagano M, Gauvreau K. *Princípios de Bioestatística*. *São Paulo*. Cengage Learning. 2012; pp. 506p.

[61] Baykal H, Yildirim HK. Application of artificial neural networks (ANNs) in wine technology. Critical Reviews in Food Science and Nutrition. 2013; 53(5): 415–421. doi: 10.1080/10408398.2010.540359 23391010

[62] Jamshidi S, Yadollahi A, Ahmadi H, Arab MM, Eftekhari M. Predicting *in vitro* culture medium macro-nutrients composition for pear rootstocks using regression analysis and neural network models. Frontiers in Plant Science. 2016; 7:274. doi: 10.3389/fpls.2016.00274 27066013 PMC4809900

